# Injurious pecking in organic turkey fattening—effects of husbandry and feeding on injuries and plumage damage of a slow- (Auburn) and a fast-growing (B.U.T.6) genotype

**DOI:** 10.1016/j.psj.2023.102746

**Published:** 2023-04-29

**Authors:** D. Haug, R. Schreiter, B. Thesing, L. Rathmann, C. Lambertz, P. Hofmann, M. Erhard, G. Bellof, E. Schmidt

**Affiliations:** ⁎ Weihenstephan-Triesdorf University of Applied Sciences, 85354 Freising, Germany; † Centre for Applied Research and Technology e.V. at the Dresden University of Applied Sciences, 01069 Dresden, Germany; ‡ Bavarian State Estates, Kitzingen, Germany; § Research Institute of Organic Agriculture (FiBL), 37213 Witzenhausen, Germany; # Bavarian State Research Center for Agriculture, Kitzingen, Germany; || Chair of Animal Welfare, Ethology, Animal Hygiene and Animal Husbandry, Department of Veterinary Sciences, LMU Munich, 80539 Munich, Germany

**Keywords:** organic turkey fattening, injurious pecking, severe feather pecking, animal welfare, plumage damage

## Abstract

Injuries and plumage damage (**PD**) are important indicators of welfare. First priority in turkey fattening is to reduce injurious pecking, which includes aggressive pecking (agonistic behavior) and additionally severe feather pecking (**SFP**) and cannibalism with their multifactorial reasons. Still, there are few studies available evaluating different genotypes for their welfare status under organic conditions. The aim of this study was to investigate the effects of genotype and husbandry with 100% organic feeding (2 variants with different riboflavin content: V1 and V2) on injuries and PD. During rearing nonbeaktrimmed male turkeys of a slow- (Auburn, *n* = 256) and fast-growing (B.U.T.6, *n* = 128) genotype were kept in 2 indoor housing systems (without environmental enrichment (**EE**) = H1−, *n* = 144 and with EE = H2+, *n* = 240). During fattening 13 animals per pen of H2+ were relocated to a free-range system (H3 MS, *n* = 104). EE included pecking stones, elevated seating platforms and silage feeding. The study included five 4-wk feeding phases. At the end of each phase, injuries and PD were scored to assess animal welfare. Injury scores ranged from 0 (=no damage) to 3 (=severe damage) and PD from 0 to 4. Injurious pecking was observed from the 8th week onward (injuries: 16.5% and PD: 31.4%). Binary logistic regression models showed that both indicators were affected by genotype (each *P* < 0.001), husbandry (each *P* < 0.001), feeding (injuries *P* = 0.004; PD *P* = 0.003), and age (each *P* < 0.001). Auburn showed less injuries and PD than B.U.T.6. H1− had the fewest injuries and PD for Auburn animals compared to H2+ or H3 MS. In summary, the use of alternative genotypes (Auburn) in organic fattening improved welfare, but keeping them in free-range systems or in husbandry with EE, does not lead to a reduction of injurious pecking. Therefore, further studies are needed with more and changing enrichment materials, further management measures, changes in housing structure, and even more intensive animal care.

## INTRODUCTION

Injuries and plumage damage (**PD**) represent major animal welfare problems in conventional and organic turkey fattening. On the one hand, pecking injuries are of considerable importance for animal welfare due to pain, suffering and damage caused to the animals, which also exposes them to an increased risk of infection that can lead to further diseases (Fiedler and König, 2005; Kulke et al., 2014). On the other hand, deaths and emergency euthanization occur because of severe pecking injuries. If these events happen frequently, there are considerable economic losses (Busayi et al., 2006; Spindler, 2007).

These behavioral disorders are subject to a multifactorial complex (Krautwald-Junghanns et al., 2017) in which genetic disposition, husbandry, management, and feeding play important roles.

To improve animal welfare, animal-related information, known as welfare indicators, is used to assess the integument condition of turkeys in addition to behavioral perceptions (Knierim et al., 2016, 2020). These welfare indicators include injuries and PD, which allow conclusions to be drawn on the welfare of the animals as well as on injurious pecking and thus on feather pecking and cannibalism.

In addition, in order to minimize the incidence of pecking, there are recommendations from the Lower Ministry of Nutrition (2019) for avoiding the occurrence of severe feather pecking (**SFP**) and cannibalism in turkeys, as well as emergency measures in the event of feather pecking and cannibalism. Nevertheless, in conventional husbandry, the beak of day-old chicks is still trimmed to minimize injuries and PD. Although this is prohibited under the German Animal Welfare Law §6 (amputation) (Federal Ministry of Justice), as it causes pain, suffering and injury to the animal, there are exceptions in subsection 3. In the case of turkeys, that means, that permission may only be granted if it is credibly demonstrated that the intervention is indispensable for the protection of the animals with regard to the intended use. However, routine implementation of this exemption should not be legitimized, but permission for beak trimming should only be granted in individual cases (Kulke et al., 2016). However, as the state of knowledge on the causes and influencing factors regarding SFP and cannibalism is still insufficient, regular stabling of beaktrimmed animals is not yet envisaged in Germany (BMEL, 2019). This is also forbidden in organic husbandry according to the Implementing Regulation (EG) No. 2018/848, but if for safety reasons or to improve the well-being or hygiene conditions of the animals, such interventions may be authorized by the competent authority on a case-by-case basis.

Injuries can be caused on the one hand by pecking or scratching by conspecifics and on the other hand by the impact of equipment (technopathies) (Krautwald-Junghanns et al., 2011, 2017; Krautwald-Junghanns and Širovnik Koščica, 2020). Various forms of pecking by conspecifics are summarized under the term injurious pecking (=repeated pecking of conspecifics at damaged house sites, resulting in bleeding and tissue damage). This term includes aggressive pecking, which is agonistic and occurs mainly in the head area (Dalton et al., 2013). This appears mainly during rank fights. In addition, there are injuries and PD because of SFP and cannibalism (Dalton et al., 2013). SFP leads to featherless areas and is defined as repeated pecking or pulling out of feathers, partial feather eating especially in the back, butt and base of the tail and leads to pain (Dalton et al., 2013). Cannibalism can result in severe bleeding injuries, with blood and tissue from living conspecifics being ingested via the beak (Dalton et al., 2013), which can ultimately lead to the death of the animals (Berk, 2002). SFP and cannibalism are distinct behavioral disorders that occur independently of each other (Keppler, 2008). Studies showed that injuries predominate in the head and neck area (Bergmann, 2006; Krautwald-Junghanns et al., 2017). Cannibalism and SFP are attributed to behavioral disorders whose causes are triggered by a multifactorial event (Spindler, 2007; Berk et al., 2013; Dalton et al., 2013; Marchewka et al., 2013). Studies by Fiedler and König (2005), Spindler et al. (2012), Kjaer and Bessei (2013), Berk et al. (2013), Schulze Bisping (2015), the Lower Ministry of Nutrition (2019), and Bartels et al. (2020) assume that the main cause is misguided foraging and exploration behavior due to an unstructured, stimulus-poor environment, which leads to lack of employment and exercise. In addition, an unsuitable stable climate, lighting, stocking density, group size, social stress, management, feeding, and endogenous influences such as age, sex, and genetic components also play a role.

According to Wartemann (2005), Schulze Bisping (2015), and Bartels et al. (2020), the prevalence of injuries due to agonistic behavior increases with age. Similarly, studies showed that PD and injuries might depend on genetics. In most cases, the lighter breed shows fewer injuries than the heavier ones (Bergmann, 2006; Busayi et al., 2006; Große Liesner, 2007; Straßmeier, 2007; Olschewsky, 2019a). In studies of Olschewsky (2019a) for example, the fewest injuries occurred in the lightest breed, Hockenhull black, with 28% compared to Hockenhull bronze and Kelly BBB (37% injuries). This is also shown by Berk and Cottin (2005) where the fast-growing B.U.T.6 had more skin lesions. In addition, according to Krautwald-Junghanns and Širovnik Koščica (2020), as well as Bircher and Schlup (1991), alternative breeds have a more pronounced comfort behavior, like the heavy cutting breeds.

Due to their body weight, the heavy breeds usually show extended lying times and reduced activity (Krautwald-Junghanns and Širovnik Koščica, 2020). Especially toward the end of fattening, the resting of B.U.T.6 increases (Bircher and Schlup, 1991). Consequently, fast-growing breeds show more PD compared to the slow-growing breeds. According to Kulke et al. (2016), a reduction in agonistic pecking actions could be demonstrated by stable structuring with elevated elements. As a rule, A-rackers or straw bales are provided in barns of organic turkey fattening. However, there is a lack of retreat possibilities for the turkeys where they can rest or hide. It would therefore be desirable to provide raised structural elements made of hygienically safe material, which can be used throughout the entire housing period (Niedersachsen, 2019). According to studies by Krautwald-Junghanns et al. (2011), Allain et al. (2013), Dalton et al. (2013), and Duggan et al. (2014), the prevalence of injuries is also very high in organic turkey farming. In principle, the first approaches to reducing injuries are known, but solutions are still being sought, because so far damage pecking can only be minimized by the measures, but not prevented. Moreover, there are still few studies evaluating slow-growing turkey breeds (with the exception of Kelly BBB) with respect to their animal welfare status under organic conditions (Olschewsky et al., 2020).

Therefore, the aim of the present study was to investigate the influence of 2 different genotypes (Auburn and B.U.T.6) with different 100% organic feeding in 3 different housing systems (indoor housing with and without environmental enrichment **(EE)**, mobile housing with free-range system) on welfare indicators (injuries and PD) to identify risk areas. The question was if under organic conditions, whether nonbeaktrimmed male Auburn turkeys or B.U.T.6 have a superior potential to lower behavioral problems, with better welfare. Second, which husbandry system ensures more animal welfare and are there feeding-related problems? Furthermore, are there effects of the structural elements that lead to the prevention of feather pecking and cannibalism?

## MATERIALS AND METHODS

### Ethical Statement

The housing conditions and all procedures performed in this study, which concerned the handling and treatment of the animals, complied with the provisions of the German Animal Welfare Act and the European Union Guidelines (2010/63/EU). The study procedures and the experimental design were reviewed and approved by the Animal Welfare Officer and Committee of the Weihenstephan-Triesdorf University of Applied Science (Permit-Number: HSWT-2022-1).

The present study is part of a larger study investigating the effects of genotype, husbandry, and feeding on behavioral abnormalities and productivity of turkeys. The focus of the present study is on turkey behavior and welfare.

### Animals, Housing, and Management

The trial was conducted from February to June 2022 at the Weihenstephan-Triesdorf University of Applied Sciences in the stables of Zurnhausen, Germany (station 1) and at the Experimental and Educational Center for Poultry Husbandry of the Bavarian State Farms in Kitzingen, Germany (station 2).

The study included five 4-wk feeding phases. The rearing phase with two 4-wk periods lasted until wk 8 of age (P1 until 4 wk of age, P2 until 8 wk of age) and the fattening phase from wk 9 to 20 (P3 until 12 wk, P4 until 16 wk, P5 until 20 wk).

On the first day of life, 384 nonbeaktrimmed male turkeys of a slow- (Auburn: Aviagen Turkeys, *n* = 256) and a fast-growing (B.U.T.6: British Aviagen Turkeys, *n* = 128) origin, each with the same parent stock, were purchased from the Moorgut Kartzfehn Turkey Breeder GmbH (Bösel, Germany). Chicks were weighed individually immediately after hatching in the hatchery. The average body weight was determined by genotype and birds within 1 standard deviation were selected for the study. The animals were allocated by body weight to the pens to achieve an equal average body weight of 1-day-old chicks in each pen.

***Rearing.*** During the rearing phase the animals were allocated to 12 pens each at the 2 stations mentioned above and kept indoors. At station 2, pens were without EE (H1−) and at station 1 with EE (H2+). H1− pens had a space allowance of 10 m^2^/pen (square layout) and H2+ pens of 6.81 m^2^/pen (elongated layout). Twelve animals per pen were housed in H1− and 20 animals per pen in H2+. This resulted in a total of 144 birds in H1− (Auburn: *n* = 96; B.U.T.6: *n* = 48) and 240 birds in H2+ (Auburn: *n* = 160, B.U.T.6: *n* = 80). As EE, pecking stones (PICKME, Witteler GmbH & Co. KG, Anröchte, Germany) were available from d 1. Pecking stones were provided as mineral supplements as well as natural means of beak abrasion. In addition, elevated seating platforms (1.25 m × 0.65 m; made of screen printing plates: Schreinerei Förg, Freising, Germany) for rearing and hiding below were installed in the pens from the first day of life. Weekly they were adjusted to the size of the turkeys. In other words, the platforms were raised at a height that allowed the animals to sit on and rest under them. In addition, they were cleaned weekly.

During rearing, with uniform postlitter management (daily), wood shavings (Equipower Allround-Span, VETRIPHARM GmbH, Hurlach, Germany) were used as litter. During the first week of age, the animals were offered attract feed on feed plates (Siepmann GmbH, Herdecke, Germany) and egg humps (Klose & Debus GbR, Ruppichteroth, Germany). Light management started with 23-h light and was reduced to 16 h by d 7. After that, there was a daily rhythm of 16-h light and 8-h dark. The light intensity was 20 lux. The temperature started at 36°C and was reduced to 18°C by the 8th week of age. Heat lamps, installed in each pen, remained until the 28th day of life. The animals were vaccinated against Newcastle Disease (Avishield ND “La Sota” Dechra Veterinary Products Deutschland GmbH, Aulendorf, Germany), Turkey Rhinotracheitis (Poulvac TRT, Zoetis Deutschland GmbH, Berlin, Germany and Nobilis Rhino, Covetrus DE GmbH, Düsseldorf, Germany) and Hemorrhagic Enteritis (Dindoral SPF, Boehringer Ingelheim Vetmedica GmbH, Ingelheim am Rhein, Germany) according to the vaccination schedule.

***Fattening.*** During fattening (from 9th to 20th week of age), animals were raised in 3 different housing systems. In addition to the 2 housing systems mentioned above, there were 2 mobile houses (agricultural modular GmbH & Co KG, Kirchham, Germany), each with 4 compartments (4 compartments/genotype), with green runout (each in total: 177 m^2^). For this, 13 birds per pen were transferred from the indoor housing with EE (H2+) to a compartment of the mobile housing (H3 MS). Seven animals per pen remained in H2+. In addition to the 2 housing enrichments (pecking stones and elevated platforms) the turkeys were supplemented with alfalfa silage (Biolandbetrieb Hans Wagner, Freinhausen, Germany), offered 3 times daily, as a third enrichment. This serves not only as supplemental feeding, but also as an enriched, natural material for additional enrichment. There were no changes in the indoor housing without EE. SoftCell (Desintec SoftCell, AGRAVIS Raiffeisen AG, Münster, Germany) was used as litter during the fattening. There was a coordinated postlitter management between the sites (weekly). Grit was given twice a week per pen (100 g; 0.6–2 mm) (DORSILIT-Kristallquarz Nr.5+7, Übelein Baustoffe GmbH, Freising, Germany). At the 20th week of age, all animals were slaughtered.

Over the whole fattening period water was also available ad libitum in height adjustable nipple or pendulum drinkers (LUBING Maschinenfabrik Ludwig Bening GmbH & Co. KG, Barnstorf, Germany). Concentrated feed was also available ad libitum in rearing (short pellets with a diameter of 2 mm) and in fattening (3 mm diameter pellets) for the animals in height adjustable feeders (Siepmann GmbH, Herdecke, Germany).

In rearing there were two 100% organic concentrate feeding variations, which differed in their riboflavin content (according to Aviagen Turkeys, 2015). Feeding variant 1 **(V1)** had a low riboflavin content of 4 mg/kg and feeding variant 2 **(V2)** had a higher content of 8 mg/kg. In the fattening period, V1 and V2 no longer differed in their riboflavin content. Both rations had a low content. In the 12th week the riboflavin level was reduced from 4 mg/kg riboflavin to 2.9 mg/kg and in the 16th and 20th week to 2.4 mg/kg.

Thus this was a 3 factorial experimental design. First there were the 2 different genotypes Auburn and B.U.T.6. The pens were randomly divided into 2 different feeding groups (V1 and V2) with 4 replicates for Auburn and 2 replicates for B.U.T.6 per site or housing system. In rearing there were the 2 housing systems (H1− and H2+) and in fattening 3 husbandry systems (to H1− and H2+ the H3 MS).

### Data Collection and Analysis

In order to assess the well-being of the turkeys, an assessment of the integument condition (injuries and PD) was scored at the end of each feeding phase (end of wk 4, 8, 12, 16, and 20). To be able to assess the criteria there were different scores. Injuries were divided into the regions wings, back, neck, and head/snood/caruncle, with a level scoring from 0 (=no damage) to 3 (=severe damage) (Supplemental Table 1: scheme according to Schulze Bisping (2015)). In the case of PD, a distinction was made between neck, back including sides of the body, wing cover, swings, and the butt with a scoring from 0 (=no damage) to 4 (=severe damage) (Supplemental Table 2: scheme according to Schulze Bisping (2015)).

Sample size was random and was 5 to 10 animals per pen (depending on stocking density). For consideration at the individual animal level, only one body side, wing or swing per animal was included in the evaluation and the part with the more serious change was always selected. Integument scoring was performed by 3 observers who completed a training period on 60 animals to determine interobserver reliability.

Daily animal control was performed and documented. Sick and injured animals were separated, cared for and excluded from the study. Mortality was recorded throughout the experiment.

### Statistical Analyses

Microsoft Excel (version 2016, Microsoft Corporation, Redmond, Washington, USA) was used to collect and prepare the data and to create diagrams. The program IBM SPSS Statistics (version 28, IBM, Chicago, IL) was used for data analysis.

For observer matching, a concordance analysis was performed to quantify the degree of agreement in the integument scores. For this purpose, the prevalence-adjusted and bias-adjusted kappa (**PABAK**) values were calculated as characteristics of the intraobserver reliability according to Gunnarsson et al. (2000). PABAK values were interpreted as follows (Landis and Koch, 1977; Kwiecien et al., 2011): <0.20 = insufficient, 0.21 to 0.40 = sufficient, 0.41 to 0.60 = moderate, 0.61 to 0.80 = good, and >0.80 = very good degree of agreement.

Injuries and PD were available as ordinally scaled characteristics in the experiment. In addition, a total score was formed for each of these characteristics from the respective individual regions and their scores by addition (Schreiter et al., 2020b). The relative proportions per score shown in the graphical figures correspond to the arithmetic mean of the respective region included. The ordinal scaled injuries and PD traits were tested univariate for effects of the husbandry type until the 8th week of age with Mann-Whitney *U* test and from the 12th week of age using the Kruskal-Wallis test (Du Prel et al., 2010). If there were significant differences between the variants, a pairwise comparison was made using the Mann-Whitney *U* test (Du Prel et al., 2010). Univariate tests for effects of genotype and dietary variants were performed using the Mann-Whitney *U* test.

Results are expressed in grouped medians for the integument traits. When scoring the integumentary characteristics, observations were grouped into classes (i.e., scores). Therefore, the grouped median was the most appropriate measure of location (Schreiter et al., 2020b).

In a second step, multiple logistic regression models were calculated with the above-mentioned integumentary traits as dependent variables and the independent variables genotype, husbandry type, feeding variant, and age as binary logistic regression (Baltes-Götz, 2012).

Nagelkerke's *R*^2^ values, which give an indication of the extent of variance in the dependent variables explained by the model, were calculated. In this context, with regard to the explanatory power of the model, Nagelkerke's *R*^2^ values ≥0.5 were considered high (Backhaus et al., 2021). For this calculation, the ordinal data scaling was transformed into a nominal scaling (Supplemental Table 3). For the injuries, 2 different nominal variants were created due to the different score range. Thus, a distinction between SFP/cannibalism and agonistic pecking is possible. Agonistic pecking occurs mainly in the head area (Dalton et al., 2013). SFP leads to featherless areas, especially in the back, butt and base of the tail (Dalton et al., 2013). Thus, there was one variant for injuries in the back and wing area and one for the neck/head/snood/caruncle area. The multicollinearity exclusion test was based on the Kendal Tau_b correlation coefficient of the factors.

Differences were considered statistically significant for *P* values of ≤0.05. Tendency was defined for 0.05 < *P* ≤ 0.1.

## RESULTS

PABAK values of 0.91 for injuries, and 0.85 for PD indicated very good interobserver reliability.

### Mortality

There were only 8 losses in total. Whereby 4 animals had to be removed from the experiment due to technopathies and 4 animals died because of unclear genesis. Of these, 7 were from the fast-growing breed and only 1 turkey from the alternative breed. No turkey was lost due to pecking injuries. Overall, there was a mortality of 2.1%.

### Injuries

The 4 scored regions were divided into 2 areas, first because the scores were distributed very differently and second to be able to distinguish between agonistic pecking and SFP. The neck/head/snood/caruncle was combined into 1 total score and the back and wings into 1 total score. There were hardly any changes in the back and wing area over the entire course (only in the 20th week on the back) (Supplemental Figure 1). In the neck/head/snood/caruncle area, the increase in age and severity had a clear influence on the injuries (Supplemental Figure 2). They occurred mainly from the 8th week of age. Most of them were found in the head area and its appendages, followed by the neck. About 14.8% of the turkeys did not show injuries in either area in the 20th week. Minor injuries (score 1) were most common throughout the entire course. However, from the 16th week onward, massive injuries (score 3) occurred, accounting for 12.5% in the head area in the 20th week.

***Genotype.*** The univariate analyses showed an influence of genetics, which were only observed in the areas of the neck and head/snood/caruncle (Table 1). B.U.T.6 showed more skin lesions (44.1%) over the entire fattening period compared to Auburn turkeys (31.8%) (Supplemental Figure 3). In the neck area, effects were visible in the 8th (*P* = 0.022), 12th (*P* = 0.013), and 16th week (*P* = 0.001). In the head area the effect was present in the 8th (*P* < 0.001), 16th (*P* = 0.002), and 20th week (*P* = 0.023). In the total score of the 2 regions, an effect of origin was evident from the 8th week (*P* < 0.001) to the 20th week (*P* = 0.025). For B.U.T.6, a peak, of injuries, was evident from wk 12 to wk 16 and for Auburn from wk 16 to week 20. B.U.T.6 had 31.3% head injuries in the 8th week and 86.5% in the 20th week, 22.9% of them have been massive injuries.

**Table 1:** Influence of genotype, husbandry system, and feeding variant on injuries in dependence of age^1^. The grouped scores are represented in the form of the grouped medians. Results of the Mann-Whitney *U* test and the Kruskal Wallis test.

Indicator/age	Genotype (G)		Husbandry system (H)^2^			Feeding variant (V)^3^		*P* values		
	Auburn	B.U.T.6	H1 -	H2 +	H3 MS	V1	V2	G	H	V
	Grouped median									
Back										
4th week	0.00	0.00	0.00	0.00		0.00	0.00	1.000	1.000	1.000
8th week	0.09	0.11	0.08	0.11		0.10	0.08	0.699	0.387	0.632
12th week	0.03	0.04	**0.07^a^**	**0.00^b^**	**0.03^ab^**	0.03	0.04	0.885	**0.033**^4^	0.515
16th week	0.02	0.03	0.05	0.03	0.00	0.02	0.03	0.813	0.072	0.690
20th week	0.09	0.16	**0.19^a^**	**0.05^b^**	**0.05^b^**	0.11	0.12	0.081	**0.002**	0.828
Wings										
4th week	0.00	0.00	0.00	0.00		0.00	0.00	1.000	1.000	1.000
8th week	0.00	0.00	0.00	0.00		0.00	0.00	1.000	1.000	1.000
12th week	0.01	0.04	**0.04^a^**	**0.00^b^**	**0.00^b^**	0.01	0.03	0.051	**0.031**	0.175
16th week	0.02	0.06	0.03	0.06	0.00	**0.06**	**0.00**	0.130	0.356	**0.044**
20th week	0.01	0.00	0.01	0.00	0.00	0.01	0.00	0.439	0.552	0.321
Neck										
4th week	0.01	0.03	0.00	0.03		0.02	0.01	0.219	0.082	0.562
8th week	**0.11**	**0.23**	**0.03**	**0.28**		0.18	0.13	**0.022**	**<0.001**	0.279
12th week	**0.20**	**0.34**	**0.18^b^**	**0.29^ab^**	**0.34^a^**	0.29	0.22	**0.013**	**0.027**	0.156
16th week	**0.36**	**0.59**	**0.22^c^**	**0.44^b^**	**0.80^a^**	0.48	0.42	**0.001**	**<0.001**	0.322
20th week	0.66	0.77	**0.35^b^**	**1.00^a^**	**1.03^a^**	0.73	0.68	0.148	**<0.001**	0.538
Head/snood/caruncle										
4th week	0.02	0.05	0.01	0.05		0.04	0.02	0.176	0.056	0.251
8th week	**0.11**	**0.33**	**0.06**	**0.32**		0.22	0.15	**<0.001**	**<0.001**	0.229
12th week	0.47	0.54	0.47	0.53	0.49	**0.57**	**0.42**	0.265	0.725	**0.023**
16th week	**0.69**	**1.03**	**0.51^a^**	**1.12^b^**	**1.14^b^**	0.88	0.75	**0.002**	**<0.001**	0.197
20th week	**1.33**	**1.59**	**0.82^b^**	**1.93^a^**	**1.79^a^**	1.52	1.33	**0.023**	**<0.001**	0.125
Total score back/wings										
4th week	0.00	0.00	0.00	0.00		0.00	0.00	1.000	1.000	1.000
8th week	0.09	0.11	0.08	0.11		0.10	0.08	0.699	0.387	0.632
12th week	0.04	0.08	**0.11^a^**	**0.00^b^**	**0.03^b^**	0.04	0.07	0.196	**0.001**	0.176
16th week	0.03	0.06	0.07	0.08	0.00	0.05	0.03	0.260	0.067	0.350
20th week	0.09	0.16	**0.20^a^**	**0.05^b^**	**0.05^b^**	0.12	0.12	0.116	**0.001**	0.982
Total score neck/head/snood/caruncle										
4th week	0.03	0.08	**0.01**	**0.08**		0.06	0.03	0.068	**0.010**	0.197
8th week	**0.22**	**0.51**	**0.08**	**0.56**		0.35	0.26	**<0.001**	**<0.001**	0.152
12th week	**0.64**	**0.84**	0.62	0.76	0.79	**0.82**	**0.61**	**0.026**	0.204	**0.012**
16th week	**1.05**	**1.67**	**0.68^b^**	**1.65^a^**	**1.96^a^**	1.42	1.20	**<0.001**	**<0.001**	0.152
20th week	**2.08**	**2.42**	**1.11^b^**	**3.00^a^**	**2.85^a^**	2.31	2.10	**0.025**	**<0.001**	0.184

1 Statistically different models for the husbandry system: for rearing in indoor housing (up to 8 wk of age) Mann-Whitney *U* and for fattening (9–20 wk) Kruskal-Wallis.

2 H1− = indoor housing without environmental enrichment; H2+ = indoor housing with environmental enrichment and silage supplementary feeding from the 9th week; H3 (MS) from 9 wk of age = mobile housing with environmental enrichment and green runout.

3 Feeding groups with different riboflavin content: V1: 4 + 8 wk: 4 mg/kg; 12 wk: 2.9 mg/kg; 16 + 20 wk: 2.4 mg/kg; V2: 4 + 8 wk: 8 mg/kg, 12 wk: 2.9 mg/kg, 16 + 20 wk: 2.4 mg/kg.

4 Bold typing and different indices (a, b, c) indicate statistically significant values.

***Husbandry.*** A husbandry influence had been recognized by univariate tests in all 4 sampled regions at all-time points (Table 1). On the back in wk 12 (*P* = 0.033) and wk 20 (*P* = 0.002). On the wings only in wk 12 (*P* = 0.031). In the 2 regions mentioned above, the injuries occurred mainly in the indoor housing without EE. In the neck region, effects were present in the 8th (*P* < 0.001), 12th (*P* = 0.027), 16th (*P* < 0.011), and 20th week (*P* < 0.001). In this skin region, the indoor housing without EE showed the least lesions. In the mobile housing a peak from the 12th to the 16th week and in the indoor housing with EE from the 16th to the 20th week had been seen. In the head area, differences were present in the 8th, 16th, and 20th week (*P* < 0.001). Significant injuries were found in the indoor housing with EE already in the 8th week, in the indoor housing without EE, in the 12th week. In the total score of neck and head, husbandry effects were evident from the 4th to the 20th week (excluded the 12th week; Supplemental Figure 4). The indoor housing without EE had the fewest injuries in both regions. In the 20th week 53.8% still had an intact skin in 1 of the 2 areas (H2+: 6.7%; H3 MS: 5.7%).

Mann-Whitney *U* test and Kruskal-Wallis test indicated that there were differences between the genotypes in the stables (Supplemental Table 3). In the overall back and wing area there have been few effects. For both genotypes injuries in this area were mainly recognizable in the indoor housing without EE.

In the neck area, husbandry effects were recognizable in the B.U.T.6 from the 8th (*P* = 0.001) to the 20th week (*P* < 0.001) and for Auburn in the 8th, 12th, and 20th week (each *P* < 0.001).

**Figure 1:**
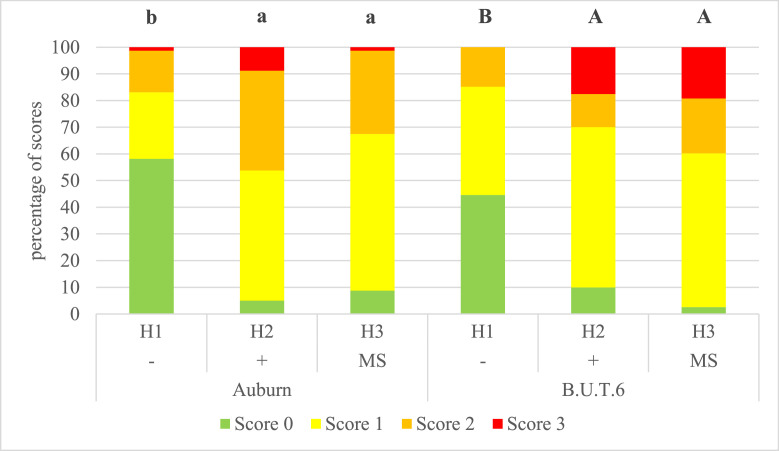
Relative percentage of scores for injuries at the neck/head/snood/caruncle region depending on genotype and husbandry system at the 20th week of life. H1− = indoor housing without environmental enrichment; H2+ = indoor housing with environmental enrichment and silage supplementary feeding from the 9th week; H3 (MS) from 9 wk of age = mobile housing with environmental enrichment and green runout; Bonitur scheme with definitions of the scores in Supplemental Table 1. Different indices within genotypes indicate significant differences between husbandry systems (*P* ≤ 0.05).

In the head area, significant differences were found in the B.U.T.6 from the 4th (*P* = 0.041) to the 20th week (*P* < 0.001) (excluded the 12th week) and in the Auburn in the 8th, 16th, and 20th week (each *P* < 0.001). For B.U.T.6 10% injuries were already found from the 4th week in the indoor housing with EE. Over the whole fattening period in the common region of the neck and head for both genotypes the least injuries were found in the indoor housing without EE. In the 20th week of age most injuries for B.U.T.6 occurred in the mobile housing (97.4%), of which 38.5% were massive injuries (score 3) in the head area. For Auburn turkeys 95% injuries were noted in the indoor housing with EE (Figure 1). Thereof 17.5% were massive injuries in the head area. However, the mobile housing showed nearly the same high values for injuries in the neck/head area in the Auburn (91.3%).

**Figure 2:**
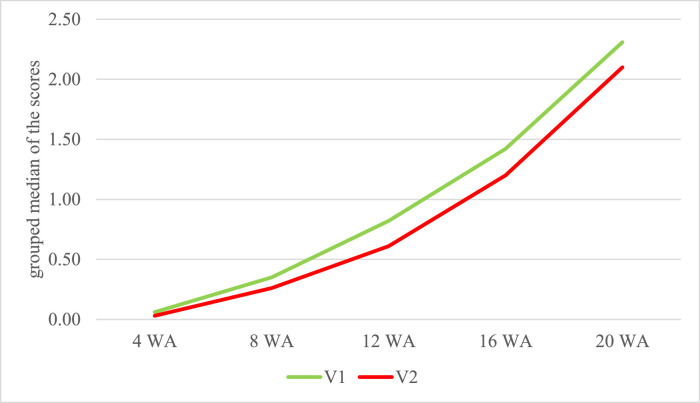
Grouped median of the scores for the injuries at the neck/head/snood/caruncle area in dependence of the feeding variant during the entire fattening period. Feeding groups with different riboflavin content: V1: 4 + 8 wk: 4 mg/kg; 12 wk: 2.9 mg/kg; 16 + 20 wk: 2.4 mg/kg; V2: 4 + 8 wk: 8 mg/kg, 12 wk: 2.9 mg/kg, 16 + 20 wk: 2.4 mg/kg; WA = week of age.

***Feeding.*** The differences of the feeding variants were recognizable in Mann-Whitney *U* tests (Table 1). However, significances could only be observed in the wing area in the 4th week (*P* = 0.044), in the head/snood/caruncle area in the 12th week (*P* = 0.023) and in the total score of the neck/head/snood/caruncle region in the 12th week of age (*P* = 0.012). In this region a higher grouped median has been seen over the fattening period in V1, which had the lower riboflavin content in the rearing period (Figure 2). There were 29.4% of injuries in V1 and 26.1% in V2.

***Logistic Regression.*** Husbandry system (*P* < 0.001), genotype (*P* < 0.001), age (*P* < 0.001), and feed variety (*P* = 0.004) influenced neck/head/snood/caruncle injuries in the significant overall logistic regression model (*P* < 0.001) with a Nagelkerke *R*^2^ of 0.518. The odds ratio's showed that compared to genotype and husbandry, feed played a lower role (Table 2). In the logistic regression model in the region back and wings the feeding was excluded from the final model (*P* = 0.975). The husbandry system (*P* < 0.001), the genotype (*P* = 0.001), and the age (*P* < 0.001) influenced this region with a Nagelkerke *R*^2^ of 0.089. Due to very low percentages of the nominal score 1 in Table 1, the explanatory power was low.

**Table 2:** Parameter estimator for the logistic regression of the characteristics injuries in the region neck/head/snood/caruncle and back/wings and the total plumage damage during the entire fattening period.

Indicator	Nagelkerke R^2^^1^	Score (1)%	Regression coefficient B	Standard error	Wald	Sig.	Exp(B)	95% confidence interval for EXP(B)	
								Lower value	Upper value
**Plumage damage**	**0.481**								
**Genotype**									
Auburn		48.7	Reference				Baseline		
B.U.T.6		69.4	1.281	0.158	66.094	<0.001	3.600	2.644	4.902
**Husbandry**					151.808	<0.001			
H1-		41.0	Reference				Baseline		
H2+		69.3	2.344	0.192	149.744	<0.001	10.424	7.161	15.174
H3 MS		69.0	0.225	0.193	1.358	0.244	1.253	0.858	1.829
**Feeding variant**									
V1		53.0	Reference				Baseline		
V2		59.4	0.427	0.144	8.819	0.003	1.532	1.156	2.030
**Age in weeks**			0.296	0.018	265.993	<0.001	1.344	1.297	1.393
Constant			-4.769	0.296	259.768	<0.001	0.008		
**Injuries neck/head/** **snood/** **caruncle**	**0.518**								
**Genotype**									
Auburn		46.6	Reference				Baseline		
B.U.T.6		61.8	0.777	0.156	24.680	<0.001	2.175	1.601	2.956
**Husbandry**					101.075	<0.001			
H1-		37.7	Reference				Baseline		
H2+		52.4	1.465	0.175	70.216	<0.001	4.329	3.073	6.100
H3 MS		86.6	1.741	0.228	58.568	<0.001	5.706	3.653	8.913
**Feeding variant**									
V1		55.0	Reference				Baseline		
V2		48.8	-0.420	0.147	8.107	0.004	0.657	0.492	0.877
**Age in weeks**			0.294	0.018	277.098	<0.001	1.341	1.296	1.389
Constant			-4.372	0.282	241.006	<0.001	0.013		
**Injuries back/wings**	**0.089**								
**Genotype**									
Auburn		5.0	Reference				Baseline		
B.U.T.6		7.9	0.617	0.240	6.581	0.010	1.853	1.157	2.969
**Husbandry**					19.071	<0.001			
H1-		8.9	Reference				Baseline		
H2+		4.2	-0.687	0.279	6.057	0.014	0.503	0.291	0.869
H3 MS		2.5	-1.766	0.446	15.663	<0.001	0.171	0.071	0.410
**Feeding variant**									
V1		6.0	Reference				Baseline		
V2		6.0	0.007	0.238	0.001	0.975	1.007	0.632	1.607
**Age in weeks**			0.098	0.023	18.350	<0.001	1.103	1.055	1.154
Constant			-3.870	0.398	94.584	<0.001	0.021		

1 explanatory power of the model: < 0.1: poor explanatory power; 0.1-0.3: modest explanatory power;0.3-0.5: moderate explanatory power; >0.5: good explanatory power

### Plumage Damage

Plumage damage was progressive with age. However, up to the 20th week, score 0 (=intact plumage) predominated with 63.1%, followed by score 1 with 28.4%. Score 4 (=high PD) did not occur during the entire fattening period (Supplemental Figure 5). Almost no or only few damages were seen in the wing area, therefore this area was not discussed further. Damages mainly affected the swings (20th week: 89.1%) and the butt (20th week: 86.7%) (Supplemental Figures 6 and 7).

***Genotype.*** The univariate analyses showed an effect of the genotype (Table 3). Plumage damage was concentrated in the swing (12th week *P* = 0.002; 20th week *P* < 0.001) and butt (8th week *P* = 0.003 to the 20th week *P* < 0.001) areas. These were already clearly visible from the 8th week. In the area of the swings an improvement was found in both genotypes in the 16th week. Therefore, there was a significant effect of origin on PD in their total score from the 8th (*P* = 0.042) to the 20th week (*P* < 0.001). Overall, Auburn showed less PD compared to B.U.T.6 (Supplemental Figures 8). In the swing and butt area, Auburn had 58.1% and B.U.T.6 had 70.8% damages of the plumage.

**Table 3:** Influence of genotype, housing system, and feed on plumage damage (in the region wings, back, neck, swings, butt, and the total score) in dependence of age^1^. The grouped scores are represented in the form of the grouped medians. Results of the Mann-Whitney *U* test and the Kruskal Wallis test.

Indicator/age	Genotype (G)		Husbandry system (H)^2^			Feeding variant (V)^3^		*P* values		
	Auburn	B.U.T.6	H1−	H2+	H3 MS	V1	V2	G	H	V
	grouped median									
Wings										
4th week	0.00	0.00	0.00	0.00		0.00	0.00	1.000	1.000	1.000
8th week	0.00	0.00	0.00	0.00		0.00	0.00	1.000	1.000	1.000
12th week	0.00	0.00	0.00	0.00	0.00	0.00	0.00	1.000	1.000	1.000
16th week	0.00	0.02	0.00	0.00	0.03	0.01	0.01	0.072	0.090	1.000
20th week	0.00	0.00	0.00	0.00	0.00	0.00	0.00	1.000	1.000	1.000
Back										
4th week	0.00	0.00	0.00	0.00		0.00	0.00	1.000	1.000	1.000
8th week	0.01	0.04	**0.00**	**0.04**		0.03	0.01	0.199	**0.024**^4^	0.175
12th week	0.02	0.03	0.03	0.00	0.03	0.04	0.01	0.548	0.255	0.099
16th week	0.02	0.04	0.03	0.06	0.01	0.01	0.04	0.306	0.180	0.252
20th week	**0.02**	**0.13**	0.03	0.11	0.08	0.06	0.07	**<0.001**	0.097	0.762
Neck										
4th week	0.00	0.00	0.00	0.00		0.00	0.00	1.000	1.000	1.000
8th week	0.08	0.09	**0.00**	**0.16**		0.06	0.10	0.758	**<0.001**	0.252
12th week	0.06	0.08	**0.01^b^**	**0.08^ab^**	**0.13^a^**	**0.10**	**0.03**	0.567	**0.003**	**0.015**
16th week	0.24	0.26	**0.00^c^**	**0.62^a^**	**0.35^b^**	0.25	0.25	0.700	**<0.001**	0.997
20th week	0.03	0.03	**0.00^b^**	**0.03^ab^**	**0.06^a^**	**0.05**	**0.00**	0.767	**0.028**	**0.008**
Swing										
4th week	0.09	0.14	0.12	0.09		0.07	0.14	0.233	0.527	0.058
8th week	1.46	1.46	**0.99**	**1.93**		1.45	1.47	0.961	**<0.001**	0.843
12th week	**1.40**	**1.60**	**1.00^c^**	**1.72^b^**	**1.96^a^**	1.46	1.50	**0.002**	**<0.001**	0.599
16th week	0.80	0.93	**0.82^b^**	**1.54^a^**	**0.32^c^**	0.79	0.90	0.156	**<0.001**	0.235
20th week	**0.89**	**1.09**	**0.97^b^**	**1.18^a^**	**0.79^c^**	**0.90**	**1.03**	**<0.001**	**<0.001**	**0.030**
Butt										
4th week	0.01	0.04	0.03	0.01		0.01	0.03	0.075	0.314	0.314
8th week	**0.53**	**0.98**	**0.10**	**1.50**		0.58	0.76	**0.003**	**<0.001**	0.193
12th week	**0.43**	**1.26**	**0.51^b^**	**0.90^a^**	**0.92^a^**	0.61	0.79	**<0.001**	**<0.001**	0.070
16th week	**0.81**	**1.18**	**0.92^b^**	**1.70^a^**	**0.29^c^**	0.84	1.05	**0.006**	**<0.001**	0.120
20th week	**0.93**	**1.62**	**1.03^b^**	**1.59^a^**	**1.17^b^**	**1.10**	**1.32**	**<0.001**	**<0.001**	**0.012**
Total score										
4th week	0.09	0.18	0.14	0.10		**0.08**	**0.17**	0.069	0.323	**0.030**
8th week	**1.80**	**2.44**	**1.09**	**3.64**		1.88	1.96	**0.042**	**<0.001**	0.352
12th week	**1.82**	**2.89**	**1.54^b^**	**2.71^a^**	**3.14^a^**	2.13	2.27	**<0.001**	**<0.001**	0.490
16th week	**1.74**	**2.38**	**1.76^b^**	**4.03^a^**	**0.96^c^**	1.82	2.13	**0.006**	**<0.001**	0.116
20th week	**1.84**	**2.82**	**2.03^b^**	**2.85^a^**	**2.09^b^**	2.13	2.33	**<0.001**	**<0.001**	0.063

1 Statistically different models for the husbandry system: for rearing in indoor housing (up to 8 wk of age) Mann-Whitney *U* and for fattening (9–20 wk) Kruskal-Wallis.

2 H1− = indoor housing without environmental enrichment; H2+ = indoor housing with environmental enrichment and silage supplementary feeding from the 9th week; H3 (MS) from 9 wk of age = mobile housing with environmental enrichment and green runout.

3 Feeding groups with different riboflavin content: V1: 4 + 8 wk: 4 mg/kg; 12 wk: 2.9 mg/kg; 16 + 20 wk: 2.4 mg/kg; V2: 4 + 8 wk: 8 mg/kg, 12 wk: 2.9 mg/kg, 16 + 20 wk: 2.4 mg/kg.

4 Bold typing and different indices (a, b, c) indicate statistically significant values.

***Husbandry.*** Recognized by univariate tests the husbandry significantly influenced the PD (Table 3) from the 8th week in 4 of the 5 bonitted regions. Noticeable here were higher grouped medians in the neck region of both genotypes in the 16th week in the mobile housing and indoor housing with EE. Highly significant influences on the swings and on the butt had been observed from the 8th to the 20th week (*P* < 0.001). The lowest damage to the swings was found in the mobile housing (although it was still highest in the 12th week) and the most damage in the indoor housing with EE. Improvement had been seen in all 3 housing types in the 16th week. The butt showed the least damage in the indoor housing without EE. In the indoor housing with EE the most PD was evident at the butt. In summary, there was a highly significant effect from the 8th week until the 20th week in the total score. Overall, the indoor housing without EE (20th week score 2 + 3 = moderate PD: 5.0%) was the husbandry with lowest PD and the indoor housing with EE (17.7%) the one with highest PD (Supplemental Figures 9).

**Figure 3:**
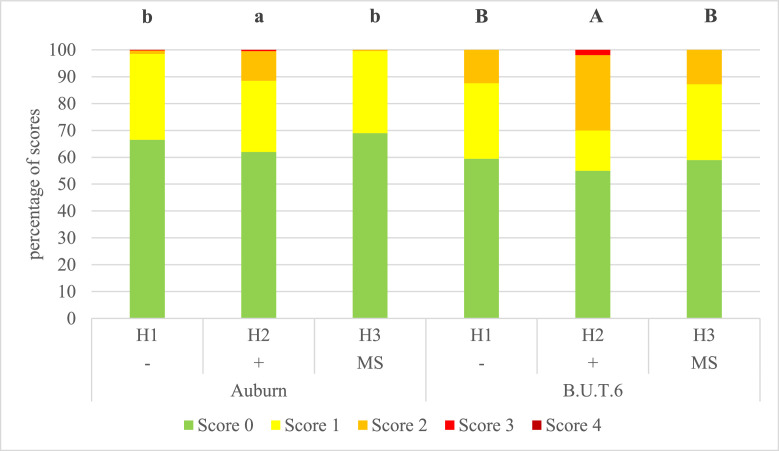
Relative percentage of scores for total plumage damage depending on genotype and husbandry system at the 20th week of life. H1− = indoor housing without environmental enrichment; H2+ = indoor housing with environmental enrichment and silage supplementary feeding from the 9th week; H3 (MS) from 9 wk of age = mobile housing with environmental enrichment and green runout; Bonitur scheme with definitions of the scores in Supplemental Table 2. Different indices within genotypes indicate significant differences between husbandry systems (*P* ≤ 0.05).

Mann-Whitney *U* test and Kruskal-Wallis test indicated that there were differences between the genotypes in the stables (Supplemental Table 4). The husbandry effect in the swing area was highly significant (*P* < 0.001) in both origins from the 8th to the 20th week. Between the genotypes, more distinct differences were visible at the butt. BU.T.6 had significantly more damages in this area compared to Auburn. For Auburn significances occurred in the 8th, 16th, and 20th week (each *P* < 0.001) and for B.U.T.6 from the 8th until the 20th week (each *P* < 0.001). Across both genotypes, the total score from wk 8 to wk 20 (each *P* < 0.001) was also highly significant. Toward the end of fattening, the mobile housing was the best housing system for Auburn (69% no PD) and the indoor housing with EE the worst (38% PD) (Figure 3). For B.U.T.6, the indoor housing with EE was also the husbandry with the most PD (45%) and the mobile housing just about the least (59% no PD).

**Figure 4:**
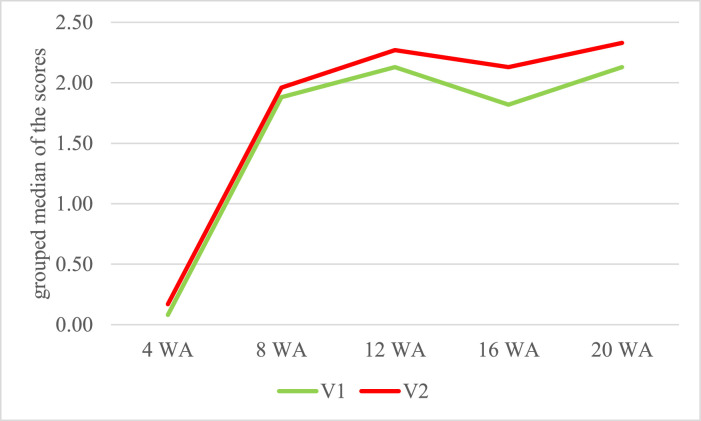
Grouped median of the scores for the total plumage damage in dependence of the feeding variant during the entire fattening period. Feeding groups with different riboflavin content: V1: 4 + 8 wk: 4 mg/kg; 12 wk: 2.9 mg/kg; 16 + 20 wk: 2.4 mg/kg; V2: 4 + 8 wk: 8 mg/kg, 12 wk: 2.9 mg/kg, 16 + 20 wk: 2.4 mg/kg; WA = week of age.

***Feeding.*** The Mann-Whitney *U* test of the feeding variants (Table 3) also showed differences. Significances were only seen in the neck area in the 12th (*P* = 0.015) and the 20th week (*P* = 0.008). In the 20th week there were differences in the swing (*P* = 0.030) and the butt (*P* = 0.012) area and in the total score in the 4th week (*P* = 0.030). A higher grouped median has been seen over the entire fattening period in V2, which had the higher riboflavin content in the rearing period (Figure 4). In V2 there were seen 28.2% PD and in V1 26.2%.

***Logistic Regression.*** In the significant logistic regression model (*P* < 0.001) with a Nagelkerke *R*^2^ of 0.481, husbandry system (*P* < 0.001), genotype (*P* < 0.001), age (*P* < 0.001), and feeding variation (*P* = 0.003) significantly influenced PD (Table 2). In summary, the feeding variants played a lower role compared to genotype and husbandry. Nevertheless, it must not be neglected that the chance for PD is 1.5 times higher with V2 than with V1.

## DISCUSSION

The study was without any disturbances and the mortality rate was low.

The aim of the study was to investigate the influence of 2 different origins (alternative breed vs. fast-growing genotype) in different housing systems and feed variants under organic conditions on the welfare indicators (injuries and PD) to identify risk areas of injurious pecking.

### Injuries

The study results showed a prevalence of 19.7% injuries of nonbeaktrimmed male turkeys. In conventional husbandry there can be an average of 32.5% injuries (Busayi et al., 2006; Große Liesner, 2007; Spindler, 2007). Bergmann (2006), Bartels et al. (2009), and Krautwald-Junghanns et al. (2011) recorded 23% injuries in organically kept turkeys. Spindler (2007) and Cottin (2004) observed fewer skin wounds in the outdoor environment. In principle, fewer injuries seem to occur in organic husbandry compared to conventional husbandry. In the study by Ermakow (2012), 22.6% of injuries occurred in conventional fattening and 14.1% in organic fattening. This was confirmed by Straßmeier (2007). Her study documented fewer injuries in organic management due to the lower stocking density. And in Olschewsky et al. (2020)), the reason for fewer injuries in organic husbandry was the smaller group size. However, there are also results with a higher prevalence of skin damage in organic fattening compared to conventional husbandry (Dressel et al., 2019). In summary, the results of the present study are comparable to those of Ermakow (2012).

In this study there were hardly any injuries in the back and wing areas (only 6% in total). As shown by Krautwald-Junghanns et al. (2011), the injuries in the back area were mainly identified as scratch wounds. Injuries in the wing area may have been caused by technopathies (e.g., when catching them for experimental data collection or when turkeys moving up and down or bump into furniture when running).

In the present clinical examinations, skin lesions were mainly found in the head area and the appendages of the snood and caruncle, as well as on the neck. The snood area was the predilection site. Studies by Bergmann (2006) and Krautwald-Junghanns et al. (2017) confirmed also injuries predominantly in the head and neck area, with the snood area being particularly affected (Krautwald-Junghanns et al., 2011; Olschewsky, 2019a). In this study, already in the 8th week, there were 17.9% injuries in the head area, which increased to 78.5% by the 20th week. Of these, 12.5% showed massive injuries (score 3) in the 20th week. In the other scored regions, a score of 3 was not found. The wounds in these areas may have been mainly attributed to mutual agonistic pecking, which is especially noticeable in the head area according to Dalton et al. (2013). The results of this study showed that in the head and neck area, the influence of age had the greatest effect on skin lesions. According to Wartemann (2005), Schulze Bisping (2015), and Bartels et al. (2020), the prevalence of injuries due to agonistic behavior increased with age. Bartels et al. (2020) assumed that younger animals are still more active, flee more easily and have less motivation against injurious pecking. In addition, the strength of the beak increases with age. In principle, turkeys begin to make threatening gestures to establish a hierarchy from the 8th week of age (Healy, 1992). Direct observations in this study showed that these already started with the 6th week of age.

***Genotype.*** Studies have identified a genetic effect on injuries. In most cases, the lighter breeds showed fewer injuries (Bergmann, 2006; Busayi et al., 2006; Große Liesner, 2007; Straßmeier, 2007; Olschewsky, 2019a). Similarly, in the study of Olschewsky (2019a), the least injuries occurred in the lightest breed (Hockenhull black) with 28% compared to Hockenhull bronze and Kelly BBB (37% injuries). The results of this study confirmed that the lighter alternative breed of Auburn (which is comparable to Hockenhull black) had a lower prevalence of injuries (17.1%) compared to B.U.T.6 (24.0%). As well as proven by Berk and Cottin (2005) where the fast-growing B.U.T.6 had more skin lesions. In the B.U.T.6 strain a high prevalence in the neck/head area was already present from the 8th week, in the present study. A large increase in lesions could have been seen from the 8th to the 12th week. This deterioration of skin condition due to injuries was seen in the Auburn only from wk 16 to wk 20. Similarly, Hockenhull black in Olschewsky et al. (2019b) showed an increase in injuries only toward the end of fattening. This may have been explained by the fact that the heavy fast-growing breeds, such as B.U.T.6, are on the one hand more precocious and therefore show agonistic behavior earlier, and on the other hand are heavier and cannot flee as quickly as the lighter alternative breeds. Auburn turkeys are considered calmer and their sexual maturity seems to start later (Fokus Tierwohl, 2022). To reduce aggressive fights, genetic selection of less aggressive lines is seen as a solution (Martrenchar et al., 1999).

***Husbandry.*** In addition, the type of husbandry had a considerable influence. According to Kulke et al. (2016), a reduction in agonistic pecking actions could be demonstrated by structuring the housing with elevated elements. Therefore, in this trial, elevated platforms were offered in the indoor housing with EE and in the mobile housing for the turkeys to retreat to and to sit on. In addition, alfalfa silage and pecking stones were offered as enriched material. Neither the offer of elevated platforms, alfalfa silage, and pecking stones in the indoor housing with EE, nor the elevated seating platforms and the green runout in the mobile housing led to a reduction in agonistic behavior, compared to the pure indoor housing. Whereas in the mobile stable 100% of the neck and head area was injured in the 20th week and 98.3% in the indoor housing with EE, only 68.4% of the neck and head area was injured in the indoor housing without EE. For both genotypes the least injuries were found in the indoor housing without EE. The most injuries for B.U.T.6 occurred in the mobile housing (77.5%) and for Auburn turkeys in the indoor housing with EE (41.6%). Similar results could also be observed in the study by Berk et al. (2017), despite the presence of enriched material, no effect of avoiding damage pecking could be achieved in the housing of beak-shortened turkeys. This resulted mainly in injuries to the appendage organs. Although EE such as hay and silage had a high acceptance rate, but in the study by Berk et al. (2013) the turkeys, which were kept with enriched material, had a higher prevalence of injuries compared to the control group. Thus, not only the lack of employment is the reason for the occurrence of injurious pecking, but numerous other factors/stressors (Bartels, 2015), which is also evident in this study.

To reduce or prevent injuries caused by technopathies the floor layout of the pens in the indoor housing with EE would have to be changed. The layout should be square and not elongated. This would allow the EE (elevated platforms and silage boxes) to be arranged in a different way. Thus, the disturbance of the resting behavior of the animals and their stress level could be reduced. In addition, more or a changing range of EE should be offered in indoor housing, as injuries start very early there. Already 10% injuries were found in this husbandry in the 4th week of age. Furthermore, direct observations showed that new people in the stable stressed the animals and led to excitement. Thus, they showed more stimulation behavior. This in turn could have led to a higher incidence of pecking. In indoor housing with EE, Auburn turkeys may have shown higher injury rates toward the end of fattening, as they are thought to mature later and are less able to avoid each other in this housing than in the free-range system.

Structures and functional areas should be installed in the runout area of the free-range system, like for example elevated seating platforms or peaked roofs. On the one hand, this offers the turkeys more variety and, on the other hand, they will probably spread out better in the runout. In the case of the B.U.T.6 animals, the injuries in the mobile housing could have occurred due to the earlier and more pronounced sexual maturity in combination with the sunlight (discussed further below) and the lazier behavior of the animals.

### Plumage Damage

Severe feather pecking usually manifests by PD in the regions of the back, butt and the neck. In addition, damage to the plumage can be caused by technopathies. Neither this studies nor Straßmeier (2007) and Olschewsky (2019a) found a progressive course and thus a reduction in plumage condition over the duration of fattening. Only in the butt area a progressive course was seen.

There was a high prevalence of PD with a total of 79.3%. Of these, Auburn had 77.6% and B.U.T.6 81.4% damages. However, no high-grade damage (score 4) occurred and score 3 only had 0.3%. Most of the damage was low-grade damage (score 1 = individual feathers are missing or damaged). Score 2 (=clear damage up to 1/2 of the plumage) was only in the butt and swing area very common. Other authors also found high prevalences. In Olschewsky (2019a) Hockenhull black had a mean of 82% PD and Kelly BBB and Hockenhull bronze 85%. Results from Bergmann (2006) showed a maximum of 23% intact plumage in organically reared turkeys. Results from Berk et al. (2006) showed 100% damages in conventionally reared B.U.T.6 birds and in Bartels et al. (2009) there were 97%. Thus, results of the present study showed similar prevalences.

Plumage damage in the wing and back area was rarely present. Damage to the back plumage could be caused by mounting on other animals, unintentional scratching injuries or technopathies.

In the neck area, most damage occurred in the 16th week of age (23.2%). Mutual pecking was assumed for PD in the neck area, which can lead to feather pecking and cannibalism (Lower Ministry of Nutrition, 2019). Comparing with the injuries, PD increased over the course of the fattening period and couldn't be linked to the PD. The damage was almost the same in both genotypes. Furthermore, indoor housing with EE and mobile housing showed the most PD. An unknown stressor seems to have caused feather pecking in the neck area between the 12th and 16th week.

The most severe damage was found on the swings and on the butt. The butt or tail area was the most severely affected. Technopathies seem to be the main cause of the damages there, because turkeys often run over the butt and swings (Schulze Bisping, 2015). In the course of fattening, the resting phases increase (Bircher and Schlup, 1991). Consequently, the turkeys run over the butt of other animals more and more often. This could indicate resting behavior and the quality of the litter (Olschewsky, 2019a). This increase in damage with age was also shown by this study results.

When catching the animals for bonitur, swing feathers were sometimes damaged, though only the tips of the wings were broken or deformed, no feathers were missing or pecked. Until the 12th week the prevalence of damaged feathers was very high, and decreased thereafter. This is partly due to the change of plumage, which is completed by the 15th week (Thiele, 2005), and partly due to the birds becoming calmer with age, resulting in less damage due to technopathies.

***Genotype.*** The investigations showed a genetic connection, which is mainly related in the swing and butt region. Auburn had a better plumage in both areas compared to the B.U.T.6. Auburn showed there a total of 58.1% and B.U.T.6 70.8% of PD. On the one hand, B.U.T.6 had a less distinctive comfort behavior compared to the Auburn turkeys. On the other hand, the high body weight and size of these turkeys played a role. As a result, they cannot reach certain regions during plumage care, or only with great difficulty, which could prevent damage due to soiling, which is also documented in Krautwald-Junghanns and Širovnik Koščica (2020). In addition, due to their weight, they usually show longer lying times and reduced activity (Krautwald-Junghanns and Širovnik Koščica, 2020). Especially toward the end of fattening, the resting of B.U.T.6 increases (Bircher and Schlup, 1991), resulting in more damage to the butt, due to other animals running over it. Direct observations in this study showed a more distinctive stimulation behavior of B.U.T.6, whereby the butt is more often fanned out and can bump more often against equipment. In addition, there might have been more damage to the swings due to the size of the birds, as they bump into equipment more often than the smaller Auburns when they were moving up and down the elevated platforms. Furthermore, the plumage color is an influencing factor that should not be ignored (Huber-Eicher and Wechsler, 1997; Fokus Tierwohl, 2022; own direct observations). Everything what glitters and shines is interesting for turkeys. Therefore, dust, dirt particles, sunlight and blood are immediately noticeable in white plumage (Huber-Eicher and Wechsler, 1997). The turkeys followed their exploring urge, which they did with their beak, and peck at these spots, which can cause damage and ultimately SFP.

***Husbandry.*** A husbandry effect was also evident in these regions. The indoor housing without EE (20th week score 2 + 3 = moderate PD: 5.0%) and the mobile housing (6.6%) were the best and the indoor housing with EE (17.7%) the worst. In summary, even for both genotypes, the indoor housing with EE was the husbandry system with the most PD and the mobile housing the one with the least. Whereas the indoor housing without EE showed almost the same amount of damage as the mobile housing. This showed that technopathies could have been mainly responsible for PD in this trial, as most damage occurred in both genotypes in the indoor housing with EE. There the elongated pen and several furnishings could have increased the risk of technopathies. The furnitures led to damage to the butt when the turkeys butt against the automatic feeders or other things during the imposing behavior with a fanned out butt. Observations showed that mainly tips were broken off at the butt, only rarely a feather was pecked. Moreover, technopathies occurred frequently in the swing area. During direct observations, it was often seen that animals bumped with the swings when jumping up or down the elevated platforms (Olschewsky, 2019a). These observations were also made in this study, where the swings on the automatic feeders, for example, were damaged or even the feathers on the swings broke off due to the bumping when jumping up or down the elevated platforms. Likewise, due to the elongated pens in this housing, the turkeys ran more frequently over the butt of resting animals. In summary, the pens have to be square and not elongated. This results in a better arrangement of the EE. This would lead to fewer injuries due to bumping and running over the butt.

In the indoor housing without EE, the least damages occurred, which is due to the fact that the animals had a square pen and fewer equipment items. This could have reduced the risk of damage caused by technopathies.

The free-range system showed a similar amount of damage as the indoor housing without EE. The least damage was found in the swing area (although it was still highest in the 12th week). In the butt area, the damage was slightly higher compared to the indoor housing without EE. Improvements in plumage condition were visible in all 3 housing types in the 16th week, whereby the greatest improvement took place in the mobile housing. The plumage change was completed by the 15th week, which could have explained the improvements. As the birds in the mobile housing and the green runout also had a softer surface and the swings do not drag over the litter during imposition, the best values were probably achieved there.

### Injuries and Plumage Damage

***Feeding.*** Likewise, a subordinate feed influence was evident for injuries and PD. The energy content of the 2 rations was similar. Differences were only present in the riboflavin content in the 4th and 8th week. Feed V1 (with the lower riboflavin content) tended to have a higher degree of injuries, but this is not statistically confirmed. Furthermore, the differences are in the marginal range (V: 29.4% and V2 26.1% injuries in the neck/head/snood/caruncle area). Perhaps one reason could have been that the animals of V1 may have had a slight riboflavin deficiency. In general, riboflavin deficiency leads to skin inflammation (Hafez and Jodas, 1997) in addition to growth depression and poor feed conversion (National Research Council, 2000). Inflammatory skin changes that may not have been visible could have caused pain and thus led to discomfort and stress for the turkeys. This, in turn, could have triggered injurious pecking. These inflammations may also have led to skin, that is more sensitive. This could favor injuries because of pecking. In summary, the feed only played a subordinate role compared to the genotype and the husbandry in this trial.

More PD was seen in V2. However, this is also not statistically confirmed. Like for injuries the differences are in the marginal range (V1 26.2% and V2 28.2% of PD). A possible better supply of V2 to the turkeys during rearing could have led to better growth on the one hand and higher activity on the other, which could cause more damage to the swings and butt due to technopathies. However, more other reasons seem to have an influence on feed, because in the overall logistic regression PD played a subordinate role compared to genotype and husbandry.

Thus, the significance of different riboflavin feeding variants has not been finally clarified and requires further studies.

Besides this, a too short feed intake is discussed as a main reason for injurious pecking. However, there are still few studies available on feed composition (except for laying hens) (Kulke et al., 2016). Therefore, most experiences result from laying hen husbandry and are tried to be applied to turkeys. According to Patt et al. (2018) and Hartini et al. (2002), an increasing raw fiber content in the feed leads to less PD and injuries in laying hen husbandry. Due to a longer feed intake and thus prolonged ingesta passage, less feather pecking occurs (Kjaer and Bessei, 2013 ). This is because more pecking strokes are necessary to pick up feed with a high raw fiber content. Consequently, the birds are longer active. In addition, pelleted feed does not satisfy the turkeys’ motivation to peck (Kjaer and Bessei, 2013 ). Similarly, Kjaer and Bessei (2013) conclude in their study that raw feathers promote intestinal passage in a similar way to raw fiber. Therefore, for Schreiter (2020a), increasing the feed intake of raw fiber in poultry is a crucial factor in reducing these behavioral disorders. This can also be seen in this study due to the low incidence of feather pecking.

However, as already mentioned, other factors also seem to be responsible for the injuries and PD.

***Normal Behavior and Stocking Density.*** It is important and necessary for the behavior of turkeys that they can develop social structures. The current group sizes in intensive husbandry do not fulfill this requirement (Krautwald-Junghanns and Širovnik Koščica, 2020). The individual can no longer distinguish between individual animals, leading to stress for the turkey. This increases the probability of conflict situations (Bircher and Schlup, 1995). In group sizes of less than 30 animals, this social structure can be formed, but aggressive pecking in particular can be seen there. Under these conditions, a strong hierarchy can be maintained (Marchewka et al., 2013). Therefore, aggressive behavior is seen as an attempt to form a robust hierarchy (Buchwalder and Huber-Eicher, 2005). In the present study, there were small group sizes of 20 to a minimum of 5 animals. Thus, the formation of social structure seems to have been one of the reasons for aggressive pecking. Kulke et al. (2022) were also able to identify agonistic interactions as a cause of pecking in small turkey flocks. As well as the size of the flock, stocking density may have played a role. However, the results of Kulke et al. (2022) did not show that low stocking density leads to fewer injuries. Although a homogeneous flock can reduce severe feather pecking, but agonistic interactions increase with the formation of the hierarchy (Fokus Tierwohl, 2022).

It is necessary for turkeys to be able to rest and sleep in order to maintain their normal biological function. That is not possible in large groups. Lying animals are often disturbed and startled due to the lack of space (Dillier, 1991). However, resting behavior is disrupted in incorrectly designed pens (e.g., elongated, as in this study) or open unstructured systems (Krautwald-Junghanns and Širovnik Koščica, 2020). This in turn leads to stress and can trigger severe feather pecking or cannibalism. Thus, the pen design in the present study should not be elongated, but square with enough space for movement without disturbing other animals.

***Light Intensity.*** Furthermore, injurious pecking is favored by high light intensity and thus by sunlight (Sherwin and Kelland, 1998; Spindler, 2007; Duggan et al., 2014), as well as by changing light conditions. According to Vehse and Ellendorff (2000), light duration and light intensity play a role in sexual maturation and the reproductive cycle. The sexual maturation of turkeys takes place above a minimum light intensity. Under short-day conditions (6L), they do not reach sexual maturity. It begins from a light length of 10.5L to 14L. Long (23L), intermittent and increasing light duration accelerates spermatogenesis (Vehse and Ellendorff, 2000). It can be concluded that due to the light intensity and the light duration in the mobile housing, the turkeys may have showed agonistic behavior earlier than the turkeys in the indoor stables. Due to higher windows compared to the indoor housing with EE, the lowest light intensity could have been found in the indoor housing without EE.

In this study, the influence of light in the exercise area nor in the indoor housing with EE could not be assessed (low-lying windows that allow sunlight into the pens compared to H1− with high windows). To clarify the influence of light, a winter trial would be necessary.

Finally, an inadequate house climate (e.g., an increased ammonia content) or temperature changes can lead to discomfort and stress and thus trigger pecking in turkeys (Lower Ministry of Nutrition, 2019).

In summary, turkeys are very sensitive animals that react immediately to changes. Therefore, if several stressors are present, injurious pecking is difficult to avoid.

The clinical examinations of this study showed especially an occurrence of agonistic pecking mainly in the snood area. Cannibalism did not occur and severe feather pecking only to a very small extent. Only in the 16th week in the neck area and in the back area, however, only in single animals and not concentrated on a certain week. In the studies by Olschewsky et al. (2020), there were just as few injuries and PD, which can be seen as positive from an animal welfare point of view and can be attributed to the small group size. In their study, there were only 3 losses due to cannibalism. Although PD and injuries occurred to a not insignificant extent, the findings did not indicate manifest problems of feather pecking and cannibalism (Olschewsky, 2019a). In beaktrimmed turkeys, Petermann (2006) found loss rates due to pecking of 8 to 10%. Kulke et al. (2022) also found an average loss rate in 2 trials of 10% in nonbeaktrimmed turkeys. The own study results show an overall loss rate of 2.1%, of which no loss was due to pecking. Therefore, it can be concluded that avoiding beak-trimming of turkeys does not always have to lead to an increased mortality rate.

## CONCLUSIONS

In this study, Auburn turkeys showed better welfare in organic husbandry in terms of injuries and PD. The male slow-growing alternative breed (Auburn) showed overall less skin lesions and PD compared to the fast-growing B.U.T.6. The mobile housing with a green runout did not improve animal welfare as such. In summer, light duration and light intensity play a decisive role in mobile housing, which mainly can lead to agonistic interaction. Comparative studies during the winter months would be helpful here. Injurious pecking caused a considerable amount of skin injuries in this study. These already occurred from the 8th week of age. The predilection site was the head and especially the snood area. Like injuries, PD occurred from the 8th week onward to a not insignificant extent, especially in the areas of butt and swings.

Although structural elements showed a high acceptance, but injurious pecking could not be prevented because of too many other influences, which led to pecking injuries.

In summary, the use of light alternative breeds (Auburn) in organic turkey fattening can be recommended to improve animal welfare and minimize the risk of pecking. However, keeping them in free-range systems with outdoor access, does not result in a reduction of injuries and PD compared to keeping them only in indoor housing or raising heavy fast-growing breeds. In summary, this may possibly be changed through improvements in management and husbandry structure. Therefore, there is a need for more and changing employment materials, already during rearing period, structuring in the outdoor area and even more intensive animal care. The individuality of the herd must always be taken into account. Further studies are needed to determine whether the welfare of alternative breeds, such as Auburn turkeys, are better in organic free-range systems due to the changes mentioned above. Likewise, the effect of feeding with different riboflavin contents must be further investigated.

## Appendix Supplementary materials

Image, application 1

## Footnotes

Supplementary material associated with this article can be found, in the online version, at doi:10.1016/j.psj.2023.102746.
