# Supplementary material for: Injurious pecking in organic turkey fattening—effects of husbandry and feeding on injuries and plumage damage of a slow- (Auburn) and a fast-growing (B.U.T.6) genotype
Source: Poult Sci. 2023 Apr 29;102(8):102746. doi: 10.1016/j.psj.2023.102746 (PMC10404662; doi:10.1016/j.psj.2023.102746)
Supplement: Supplementary file 1 [file mmc1.docx]

**Supplemental Material**

**Table S1:** Scoring scheme of injuries in the area of neck, back incl. body sides and wings (scores from 0 to 3) and the region head/snood/caruncle (scores from 0 to 3) to assess the integumentary state

| **Criteria/Score** | | **Definition** |
| --- | --- | --- |
| **Injuries of the neck, back, wings^1^** | |  |
| **0** | intact skin | no injuries |
| **1** | low grade injuries | skin injuries < 2cm |
| **2** | moderate injuries | skin injuries 2-8cm |
| **3** | massive injuries | skin injuries > 8cm |
| **Injuries of the head/cone/wattles^2^** | |  |
| **0** | intact skin | no injuries |
| **1** | low grade injuries | injury(s) < 0.5 cm and/or  haematoma present and/or  frontal snood < 25 % injured/scabbed |
| **2** | moderate injuries | injury(s) 0.5 - 2 cm and/or  frontal snood 25 - 50 % injured/scabbed |
| **3** | massive injuries | injury(s) > 2 cm and/or  frontal snood > 50 % injured/scabbed |

*^1^Scheme according to Schulze-Bisping (2015)*

^2^Sc*heme modified according to Schulze-Bisping (2015)*

**Table S2**: Scoring scheme of plumage damage in the area of neck, back incl. body sides and wing coverts (from score 0 to 4) and in the areas swings and butt (scores from 0 to 4) to assess the integumentary state

| **Criteria/Score** | | | **Definition** |
| --- | --- | --- | --- |
| **Plumage damage neck,back, wings** | | |  |
| **0** | intact plumage | complete, attached plumage | |
| **1** | low grade plumage damage | single feathers missing or damaged (pecked/broken/teased) | |
| **2** | moderate plumage damage | several feathers missing or damaged (pecked/broken/dishevelled) and/or  one/several featherless areas with feather attachment < 2 cm | |
| **3** | moderate plumage damage | many feathers missing or damaged (pecked/broken/teethed) and/or  one/several featherless areas with feather attachment 2-8 cm | |
| **4** | high grade plumage damage | feathers are missing or damaged (pecked/broken/teethed) over the whole area and/or  one/several featherless areas with feather attachment > 8 cm | |
| **Plumage damage swings and butt** | |  | |
| **0** | intact plumage | intact plumage | |
| **1** | low grade plumage damage | individual feathers missing or damaged at the tips (pecked/broken/teared) | |
| **2** | moderate plumage damage | clear damage up to 1/2 of the plumage | |
| **3** | moderate plumage damage | clear damage up to 2/3 of the plumage | |
| **4** | high grade plumage damage | clear damage at > 2/3 of the plumage | |

*Scheme supplemented according to Schulze-Bisping (2015)*

**Table S3**: Overview of the transformation of ordinal bonitour scores into a nominal scale for the application of binary logistic regression

| **Criteria** | **Score**  **(ordinal)** | **Category**  **(nominal)** | **Description of the nominal categories** |
| --- | --- | --- | --- |
| Plumage damage |  |  |  |
|  | 0-1 | 0 | no plumage damage |
|  | 2-8 | 1 | damage of the plumage |
| Back/wings injuries |  |  |  |
|  | 0 | 0 | no injuries |
|  | 1-3 | 1 | skin injuries |
| Neck/head/snood/  caruncle injuries |  |  |  |
|  | 0 | 0 | no injuries |
|  | 1-6 | 1 | skin injuries |

**Figure S1:** Relative percentage of scores for injuries at the neck/head/snood/caruncle region over the entire fattening period.

*WA = week of age; Bonitur scheme with definitions of the scores in supplemental table 1*

**Figure S2:** Relative percentage of scores for injuries at the back and wings regions over the entire fattening period.

*WA = week of age; Bonitur scheme with definitions of the scores in supplemental table 1*

**Figure S3:** Relative percentage of scores for injuries at the neck/head/snood/caruncle region depending on the genotype over the entire fattening period.

*WA = week of age; Bonitur scheme with definitions of the scores in supplemental table 1*

**Figure S4:** Relative percentage of scores for injuries at the neck/head/snood/caruncle region depending on the husbandry system at the 20^th^ week of life.

*H1 - = indoor housing without environmental enrichment; H2 + = indoor housing with environmental enrichment and silage supplementary feeding from the 9^th^ week; H3 (MS) from 9 weeks of age = mobile housing with environmental enrichment and green runout;*

*WA = week of age; Bonitur scheme with definitions of the scores in supplemental table 1*

**Table S3:** Influence of the husbandry system on injuries on dependece of age and genotype. The grouped scores are represented in the form of the grouped medians. Results of the Mann-Whitney-U-test and the Kruskal Wallis test^1^.

| **Indicator/age** | **Husbandry system (H)^2^** | | | | | | | | | **p-value** | |
| --- | --- | --- | --- | --- | --- | --- | --- | --- | --- | --- | --- |
|  | **Auburn** | | | | **B.U.T.6** | | | | | **Auburn** | **B.U.T.6** |
|  | **H1**  **-** | | **H2**  **+** | **H3**  **MS** | **H1**  **-** | **H2**  **+** | | **H3**  **MS** | |  |  |
|  | **grouped median** | | | | | | | | |  |  |
| Back |  |  | |  |  | | | | |  |  |
| 4^th^ week | 0.00 | | 0.00 |  | 0.00 | | 0.00 | |  | 1.000 | 1.000 |
| 8^th^ week | 0.08 | | 0.10 |  | 0.08 | | 14.00 | |  | 0.616 | 0.437 |
| 12^th^ week | 0.06 | | 0.00 | 0.03 | 0.08 | | 0.00 | | 0.03 | 0.134 | 0.240 |
| 16^th^ week | 0.04 | | 0.04 | 0.00 | 0.08 | | 0.00 | | 0.00 | 0.447 | 0.087 |
| 20^th^ week | 0.14 | | 0.03 | 0.05 | **0.32^a^** | | **0.10^ab^** | | **0.05^b^** | 0.077 | **0.008^3^** |
| Wings |  | |  |  |  | |  | |  |  |  |
| 4^th^ week | 0.00 | | 0.00 |  | 0.00 | | 0.00 | |  | 1.000 | 1.000 |
| 8^th^ week | 0.00 | | 0.00 |  | 0.00 | | 0.00 | |  | 1.000 | 1.000 |
| 12^th^ week | 0.01 | | 0.00 | 0.00 | 0.11 | | 0.00 | | 0.00 | 0.549 | **0.030** |
| 16^th^ week | 0.04 | | 0.00 | 0.00 | 0.05 | | 0.17 | | 0.00 | 0.577 | 0.153 |
| 20^th^ week | 0.01 | | 0.00 | 0.00 | 0.00 | | 0.00 | | 0.00 | 0.607 | 1.000 |
| Neck |  | |  |  |  | |  | |  |  |  |
| 4^th^ week | 0.00 | | 0.01 |  | 0.00 | | 0.05 | |  | 0.317 | 0.155 |
| 8^th^ week | **0.00** | | **0.23** |  | **0.08** | | **0.38** | |  | **<0.001** | **0.001** |
| 12^th^ week | 0.18 | | 0.23 | 0.23 | **0.18^b^** | | **0.39^ab^** | | **0.46^a^** | 0.674 | **0.032** |
| 16^th^ week | **0.15^c^** | | **0.40^b^** | **0.67^a^** | **0.37^b^** | | **0.52^ab^** | | **0.85^a^** | **<0.001** | **<0.001** |
| 20^th^ week | **0.31^b^** | | **1.05^a^** | **1.03^a^** | **0.43^b^** | | **0.89^a^** | | **1.03^a^** | **<0.001** | **<0.001** |
| Head/snood/caruncle |  | |  |  |  | |  | |  |  |  |
| 4^th^ week | 0.01 | | 0.03 |  | **0.00** | | **0.10** | |  | 0.561 | **0.041** |
| 8^th^ week | **0.03** | | **0.20** |  | **0.13** | | **0.57** | |  | **<0.001** | **<0.001** |
| 12^th^ week | 0.49 | | 0.48 | 0.40 | 0.44 | | 0.63 | | 0.58 | 0.660 | 0.302 |
| 16^th^ week | **0.43^b^** | | **1.03^a^** | **0.97^a^** | **0.67^b^** | | **1.23^a^** | | **1.28^a^** | **<0.001** | **<0.001** |
| 20^th^ week | **0.77^b^** | | **1.97^a^** | **1.51^a^** | **0.93^b^** | | **1.83^a^** | | **2.17^a^** | **<0.001** | **<0.001** |
| Total score back/wings |  | |  |  |  | |  | |  |  |  |
| 4^th^ week | 0.00 | | 0.04 |  | 0.00 | | 0.00 | |  | 1.000 | 1.000 |
| 8^th^ week | 0.08 | | 0.10 |  | 0.08 | | 0.14 | |  | 0.616 | 0.437 |
| 12^th^ week | 0.08 | | 0.00 | 0.03 | **0.19^a^** | | **0.00^b^** | | **0.03^b^** | 0.077 | **0.008** |
| 16^th^ week | 0.05 | | 0.04 | 0.00 | 0.10 | | 0.17 | | 0.00 | 0.291 | 0.137 |
| 20^th^ week | **0.15** | | **0.03** | **0.05** | **0.32^a^** | | **0.10^ab^** | | **0.05^b^** | **0.048** | **0.008** |
| Total score neck/head/  snood/caruncle |  | |  |  |  | |  | |  |  |  |
| 4^th^ week | 0.01 | | 0.04 |  | **0.00** | | **0.15** | |  | 0.313 | **0.011** |
| 8^th^ week | **0.03** | | **0.42** |  | **0.18** | | **0.93** | |  | **<0.001** | **<0.001** |
| 12^th^ week | 0.64 | | 0.67 | 0.59 | 0.60 | | 1.00 | | 1.03 | 0.860 | **0.047** |
| 16^th^ week | **0.56^b^** | | **1.55^a^** | **1.74^a^** | **0.96^b^** | | **1.79^a^** | | **2.16^a^** | **<0.001** | **<0.001** |
| 20^th^ week | **0.96^b^** | | **3.06^a^** | **2.54^a^** | **1.35^b^** | | **2.80^a^** | | **3.17^a^** | **<0.001** | **<0.001** |

*^1^Statistically different models for the husbandry system: for rearing in indoor housing (up to 8 weeks of age) Mann Withney U and for fattening (9-20 weeks) Kruskal Wallis*

*^2^H1 - = indoor housing without environmental enrichment; H2 + = indoor housing with environmental enrichment and silage supplementary feeding from the 9^th^ week; H3 (MS) from 9 weeks of age = mobile housing with environmental enrichment and green runout*

*^3^Bold typing and different indices (a, b, c) indicate statistically significant values*

**Figure S5:** Relative percentage of scores for the total plumage damage of all scored regions (wing, back, neck, swing, butt) over the entire fattening period.

*WA = week of age; Bonitur scheme with definitions of the scores in supplemental table 2*

**Figure S6:** Relative percentage of scores for plumage damage at the swings over the entire fattening period.

*WA = week of age; Bonitur scheme with definitions of the scores in supplemental table 2*

**Figure S7:** Relative percentage of scores for plumage damage at the butt over the enitre fattening period.

*WA = week of age; Bonitur scheme with definitions of the scores in supplemental table 2*

**Figure S8:** Relative percentage of scores for the total plumage damage depending on the genotype over the enitre fattening period.

*WA= week of age; Bonitur scheme with definitions of the scores in supplemental table 2*

**Figure S9:** Relative percentage of scores for the total plumage damage in depending on the husbandry system during the entire fattening period.

*H1 - = indoor housing without environmental enrichment; H2 + = indoor housing with environmental enrichment and silage supplementary feeding from the 9^th^ week; H3 (MS) from 9 weeks of age = mobile housing with environmental enrichment and green runout;*

*WA= week of age; Bonitur scheme with definitions of the scores in supplemental table 2*

**Table S4**: Influence of the husbandry system on plumage damage in dependece of age and genotype. The grouped scores are represented in the form of the grouped medians. Results of the Mann-Whitney-U-test and the Kruskal Wallis test^1^.

| **Indicator/age** | **Husbandry system (H)^2^** | | | | | | | | | **p-value** | |
| --- | --- | --- | --- | --- | --- | --- | --- | --- | --- | --- | --- |
|  | **Auburn** | | | | **B.U.T.6** | | | | | **Auburn** | **B.U.T.6** |
|  | **H1**  **-** | | **H2**  **+** | **H3**  **MS** | **H1**  **-** | **H2**  **+** | | **H3**  **MS** | |  |  |
|  | **grouped median** | | | | | | | | |  |  |
| Wings |  |  | |  |  | | | | |  |  |
| 4^th^ week | 0.00 | | 0.00 |  | 0.00 | | 0.00 | |  | 1.000 | 1.000 |
| 8^th^ week | 0.00 | | 0.00 |  | 0.00 | | 0.00 | |  | 1.000 | 1.000 |
| 12^th^ week | 0.00 | | 0.00 | 0.00 | 0.00 | | 0.00 | | 0.00 | 1.000 | 1.000 |
| 16^th^ week | 0.00 | | 0.00 | 0.00 | 0.00 | | 0.00 | | 0.05 | 1.000 | 0.199 |
| 20^th^ week | 0.00 | | 0.00 | 0.00 | 0.00 | | 0.00 | | 0.00 | 1.000 | 1.000 |
| Back |  | |  |  |  | |  | |  |  |  |
| 4^th^ week | 0.00 | | 0.00 |  | 0.00 | | 0.00 | |  | 1.000 | 1.000 |
| 8^th^ week | 0.00 | | 0.03 |  | 0.00 | | 0.08 | |  | 0.156 | 0.079 |
| 12^th^ week | 0.03 | | 0.00 | 0.05 | 0.08 | | 0.00 | | 0.00 | 0.166 | 0.074 |
| 16^th^ week | 0.04 | | 0.04 | 0.00 | 0.05 | | 0.08 | | 0.03 | 0.308 | 0.444 |
| 20^th^ week | 0.00 | | 0.05 | 0.05 | 0.08 | | 0.22 | | 0.14 | 0.156 | 0.408 |
| Neck |  | |  |  |  | |  | |  |  |  |
| 4^th^ week | 0.00 | | 0.00 |  | 0.00 | | 0.00 | |  | 1.000 | 1.000 |
| 8^th^ week | **0.00** | | **0.15** |  | **0.00** | | **0.18** | |  | **<0.001^3^** | **0.006** |
| 12^th^ week | **0.00^b^** | | **0.07^ab^** | **0.16^a^** | 0.03 | | 0.11 | | 0.10 | **0.003** | 0.323 |
| 16^th^ week | **0.00^c^** | | **0.64^a^** | **0.32^b^** | **0.00^b^** | | **0.57^a^** | | **0.37^a^** | **<0.001** | **<0.001** |
| 20^th^ week | **0.00^b^** | | **0.03^ab^** | **0.08^a^** | 0.00 | | 0.05 | | 0.05 | **0.047** | 0.382 |
| Swing |  | |  |  |  | |  | |  |  |  |
| 4^th^ week | 0.10 | | 0.08 |  | 0.15 | | 0.13 | |  | 0.577 | 0.747 |
| 8^th^ week | **0.99** | | **1.93** |  | **1.00** | | **1.93** | |  | **<0.001** | **<0.001** |
| 12^th^ week | **1.00^c^** | | **1.63^b^** | **1.93^a^** | **1.00^b^** | | **1.89^a^** | | **2.00^a^** | **<0.001** | **<0.001** |
| 16^th^ week | **0.76^b^** | | **1.48^a^** | **0.16^c^** | **0.93^b^** | | **1.67^a^** | | **0.49^c^** | **<0.001** | **<0.001** |
| 20^th^ week | **0.95^a^** | | **1.03^a^** | **0.65^b^** | **1.03^b^** | | **1.45^a^** | | **0.94^b^** | **<0.001** | **<0.001** |
| Butt |  | |  |  |  | |  | |  |  |  |
| 4^th^ week | 0.01 | | 0.00 |  | 0.05 | | 0.03 | |  | 0.317 | 0.559 |
| 8^th^ week | **0.03** | | **1.33** |  | **0.25** | | **1.76** | |  | **<0.001** | **<0.001** |
| 12^th^ week | 0.35 | | 0.57 | 0.43 | **0.83^b^** | | **1.54^a^** | | **1.53^a^** | 0.099 | **<0.001** |
| 16^th^ week | **0.69^b^** | | **1.68^a^** | **0.23^c^** | **1.41^b^** | | **2.00^a^** | | **0.35^c^** | **<0.001** | **<0.001** |
| 20^th^ week | **0.78^b^** | | **1.38^a^** | **0.80^b^** | **1.53^b^** | | **2.00^a^** | | **1.51^b^** | **<0.001** | **<0.001** |
| Total score |  | |  |  |  | |  | |  |  |  |
| 4^th^ week | 0.11 | | 0.08 |  | 0.20 | | 0.15 | |  | 0.417 | 0.559 |
| 8^th^ week | **1.01** | | **3.45** |  | **1.25** | | **3.94** | |  | **<0.001** | **<0.001** |
| 12^th^ week | **1.35^b^** | | **2.25^a^** | **2.61^a^** | **1.92^b^** | | **3.52^a^** | | **3.63^a^** | **<0.001** | **<0.001** |
| 16^th^ week | **1.49^b^** | | **3.84^a^** | **0.73^c^** | **2.37^b^** | | **4.36^a^** | | **1.27^c^** | **<0.001** | **<0.001** |
| 20^th^ week | **1.74^b^** | | **2.43^a^** | **1.58^b^** | **2.65^b^** | | **3.69^a^** | | **2.61^b^** | **<0.001** | **<0.001** |

*^1^Statistically different models for the husbandry system: for rearing in indoor housing (up to 8 weeks of age) Mann-Withney-U and for fattening (9-20 weeks) Kruskal Wallis*

*^2^H1 - = indoor housing without environmental enrichment; H2 + = indoor housing with environmental enrichment and silage supplementary feeding from the 9^th^ week; H3 (MS) from 9 weeks of age = mobile housing with environmental enrichment and green runout*

*^3^Bold typing and different indices (a, b, c) indicate statistically significant values*
